# Photocrosslinkable gelatin-treated dentin matrix hydrogel as a novel pulp capping agent for dentin regeneration: I. synthesis, characterizations and grafting optimization

**DOI:** 10.1186/s12903-023-03236-z

**Published:** 2023-08-04

**Authors:** Eman M. Sedek, Elbadawy A. Kamoun, Nehal M. El-Deeb, Sally Abdelkader, Amal E. Fahmy, Samir R. Nouh, Nesma Mohamed Khalil

**Affiliations:** 1 Dental Biomaterials Department, Faculty of Dentistry, Alexandria University, Champolion St., Azarita, Alexandria, Egypt; 2 Polymeric Materials Research Department, Advanced Technology and New Materials Research Institute, City of Scientific Research and Technological Applications (SRTA-City), New Borg Al-Arab City, Alexandria, 21934 Egypt; 3 Nanotechnology Research Center (NTRC), The British University in Egypt, El-Shreouk City, Cairo, Egypt; 4 Biopharmaceutical Products Research Department, Genetic Engineering and Biotechnology Research Institute, City of Scientific Research and Technological Applications (SRTA-City, Alexandria, New Borg El-Arab City, Egypt; 5 Surgery Department, Faculty of Veterinary Medicine, Alexandria University, Alexandria, Egypt; 6 Oral Biology Department, Faculty of Dentistry, Alexandria University, Alexandria, Egypt

**Keywords:** Gelatin, Riboflavin, Glycine, Dentin matrix, Injectable scaffold, Dentin regeneration, Pulp capping

## Abstract

**Background:**

In recent years, treated dentin matrix (TDM) has been introduced as a bioactive hydrogel for dentin regeneration in DPC. However, no study has introduced TDM as a photocrosslinkable hydrogel with a natural photoinitiating system. Therefore, the present study aimed to explore the synthesis, characterizations and grafting optimization of injectable gelatin- glycidyl methacrylate (GMA)/TDM hydrogels as a novel photocrosslinkable pulp capping agent for dentin regeneration.

**Methods:**

G-GMA/TDM hydrogel was photocrosslinked using a new two-component photoinitiating system composed of riboflavin as a photoinitiator under visible light and glycine as a first time coinitiator with riboflavin. The grafting reaction conditions of G-GMA/TDM *e.g*. GMA concentration and reaction time were optimized. The kinetic parameters *e.g.* grafting efficiency (GE) and grafting percentage (GP%) were calculated to optimize the grafting reaction, while yield (%) was determined to monitor the formation of the hydrogel. Moreover, G-GMA/TDM hydrogels were characterized by swelling ratio, degradation degree, and cytotoxicity. The instrumental characterizations *e.g.* FTIR, ^1^H-NMR, SEM and TGA, were investigated for verifying the grafting reaction. Statistical analysis was performed using F test (ANOVA) and Post Hoc Test (*P* = 0.05).

**Results:**

The grafting reaction dramatically increased with an increase of both GMA concentration and reaction time. It was realized that the swelling degree and degradation rate of G-GMA/TDM hydrogels were significantly reduced by increasing the GMA concentration and prolonging the reaction time. When compared to the safe low and moderate GMA content hydrogels (0.048, 0.097 M) and shorter reaction times (6, 12, 24 h), G-GMA/TDM with high GMA contents (0.195, 0.391 M) and a prolonged reaction time (48 h) demonstrated cytotoxic effects against cells using the MTT assay. Also, the morphological surface of G-GMA/TDM freeze-dried gels was found more compacted, smooth and uniform due to the grafting process. Significant thermal stability was noticed due to the grafting reaction of G-GMA/TDM throughout the TGA results.

**Conclusions:**

G-GMA/TDM composite hydrogel formed by the riboflavin/glycine photoinitiating system is a potential bioactive and biocompatible system for in-situ crosslinking the activated-light pulp capping agent for dentin regeneration.

**Supplementary Information:**

The online version contains supplementary material available at 10.1186/s12903-023-03236-z.

## Background

Hydrogels are three-dimensional hydrophilic polymeric networks capable of swelling and retaining a large volume of water [1]. Lately, in situ hydrogel formation has been extensively employed as a carrier for biomedical applications because of its simple formation in any required shape, the lack of organic solvents or chemicals used, and the fact that the viscoelasticity of the in situ hydrogel is similar to the extracellular matrix (ECM), which can mimic the three-dimensional microenvironment of cells, support cell attachment, and induce cell proliferation and differentiation [2, 3]. Thus, hydrogels formed in situ met all typical physiological requirements that could meet the general requirements of a scaffold [1].

Surprisingly, in situ forming hydrogels were widely used for a variety of medical applications, including tissue engineering, medication delivery, wound healing, and tissue regeneration [4–6]. Nowadays, gelatin-derived hydrogels are widely employed as interesting regenerative scaffolds for mimicking biotissues, creating beds for tissue healing and drug administration, *etc.* [7]. Gelatin (G) is a natural polymer and one of the derivatives of collagen that could be an appropriate component of scaffolds due to its low immunogenicity and similarity to the extracellular matrix (ECM). Moreover, it improves cell attachment and biodegradability of scaffolds and induces cell proliferation [8]. In recent years, gelatin has been used in interaction with human dental pulp stem cells for bone tissue engineering [9], dentin-pulp complex tissue engineering [10], dentin regeneration [11] , and Dental Follicle Stem Cells for tooth root regeneration [12].

Dentin matrix obtained from extracted teeth has been shown to act as a biocompatible scaffold for attachment, proliferation and differentiation of dental pulp stem cells into odontoblast-like cells [13]. The derived dentin matrix had no bearing on its infectivity because of the avascular and acellular nature of the dense collagenous matrix, thus rendering the allogenic human dentin matrix non-immunogenic [14]. Treated dentin matrix (TDM), as an acellular material, has been thought to be promise in dentin regeneration. Previous research suggested that treated dentin matrix (TDM) extracts contained a variety of extracellular matrix molecules, including dentin sialoprotein (DSP), transforming growth factor-β, decorin, biglycan, and dentin matrix protein 1 (DMP-1) that mediated cell proliferation and odontogenic differentiation [15, 16]. Furthermore, TDM was able to activate stem cells in pulp tissues to engage in pulp healing by promoting the production of restorative dentin, resulting in entire dentin regeneration (dentin, predentin, and the odontoblast layer) [6, 17, 18].

Photo-crosslinking to form hydrogels has attracted considerable interest in the field of tissue engineering. The reason for this interest is that gel constructs have similar water contents to the extracellular matrix and thus allow for efficient nutrient transport, which is important for maintaining cell viability, as well as contributing to biocompatibility by reducing mechanical irritation to the surrounding tissue [19–21]. The photoinitiators used in visible light mediated hydrogelation have been somewhat limited due to cytotoxicity and solubility issues [22]. Potential sources of biocompatible photoinitiators include free-radical-generating molecules that are naturally found in biological systems. Riboflavin, also known as vitamin B2, is naturally occurring in the body, non-toxic and absorbs light strongly between 330–470 nm, making it an attractive alternative to the current synthetic photoinitiators. Riboflavin is a type II photoinitiator; hence, a coinitiator is required as a proton donor to start the polymerization reaction [23]. For the first time, glycine is combined with riboflavin as a safe and effective natural coinitiator rather than an amine initiator. Glycine is the simplest of amino acids and an integral component of critical biological molecules as well as a central component of many metabolic reactions [24].

The aim of this study is to synthesize gelatin- glycidyl methacrylate/treated dentin matrix hydrogel and optimize the grafting reaction through grafting conditions such as GMA concentration and reaction time. The efficiency of the photopolymerization system has been assessed in terms of its influence on the entire formed hydrogel properties e.g. swelling ratio, degradation degree, hydrogel morphology and thermal Stability. Moreover, the kinetic parameters of copolymers and cytotoxicity were tested.

## Materials and methods

### Materials

Gelatin from porcine skin (type A, 300 bloom corresponding to a molecular weight range of 50 to 100 kDa), Glycidyl methacrylate (GC, ≥97.0%) and triethylamine were supplied from Sigma-Aldrich Chemie GmbH (Steinheim, Germany). Riboflavin and glycine were taken up from Sigma-Aldrich (St. Louis, MO, USA). Dimethyl Sulfoxide (DMSO) was obtained from Fluka Chemie, Germany. Dialysis tubing cellulose membrane (Mwt cut-off 14000, average diameter 16 mm) was obtained from Merck, Germany. A blue light-emitting diode lamp (LED-lamp) (Bluephase, Ivoclar Vivadent, Amhest, NY, USA) was used for irradiation at λmax 460 nm and a light intensity of 1100 mW/cm2. The distance between the irradiation light source and the sample was almost 0 cm. The irradiation time was *ca.* ≥ 50 seconds.

### Methods

#### Grafting reaction of G-GMA copolymer:

##### (Different GMA concentrations)

Gelatin (2 g) was dissolved in 150 mL of DMSO under a magnetic stirring speed of 300 rpm at 50 °C for 3 h. After the mixture was completely homogenized, the pH of the mixture was adjusted to 9 with triethylamine. A definite amount of glycidyl methacrylate was added to the gelatin solution to obtain the following molar ratios (Gelatin/GMA) ~ (1:0.048, 1:0.097, 1:0.195, 1:0.391 M). The mixture solution was preserved under vigorous stirring for 24 h at 60 °C. Next, the solution was dialyzed for 3 days at 40 °C followed by freeze drying and storage at -20 °C until further use [7, 25, 26].

##### (Different reaction time)

Other groups of gelatin (2 g) were dissolved in 150 mL of DMSO at a stirring speed of 300 rpm at 50°C for 3 h. After the mixture was completely homogenized, the pH of the mixture was adjusted to 9 with triethylamine. Then, 2 mL (1:0.097 M) of glycidyl methacrylate was mixed into the solution by constant and vigorous stirring at 60 °C for different reaction times (6 h, 12 h, 24 h and 48 h). Next, the solution was dialyzed for 3 days at 40 °C followed by freeze drying and storage at -20 °C until further use [7, 25].

#### Preparation of treated dentin matrix powder

Forty freshly extracted sound mandibular and maxillary first and second molars were collected from recently discarded 1-2 year -old male dog jaws obtained from the animal house of the Medical Research Institute, Alexandria University, for experimental reasons using a TDM treatment protocol under aseptic conditions. The teeth upon removal were stored in saline and then thoroughly washed with deionized water and immersed in 70% ethanol for 20 h, then rinsed. The crown and cementum were eliminated using a high-speed fissure carbide bur under water coolant. Pulp tissue was also removed using endodontic files [4, 27].

The roots were perforated to adequately perfuse the EDTA solution. The samples were treated with 17% EDTA, 10% EDTA and 5% EDTA for 10 min, 10 min and 5 min, respectively. To eliminate the organic content, the roots were immersed in isopropanol for 2 h and then rinsed with deionized water [27]. Samples were then stored in a saline solution in a refrigerator until milling. Eventually, the resultant TDM was grounded to a fine powder using grinding apparatus, and the resultant powder was sieved to obtain a particle size of less than 76 µm. According to Chen et al*.* [28], who used TDM with a <76 μm particle size as a pulp capping agent for dentin regeneration, this is an acceptable range for introducing TDM in a unique form other than powder-like (such as paste or hydrogel), as well as allowing injectability of the constructed scaffold. The obtained powder was then maintained in sterile phosphate-buffered saline (PBS) with 100 units/ml penicillin and 100 mg/ml streptomycin for 72 h, washed in sterile deionized water for 20 min in an ultrasonic cleaner and stored at 4 °C [14, 29].

#### Crosslinking of gelatin-GMA /TDM hydrogel under visible light irradiation

The freeze dried gelatin - glycidyl methacrylate (G-GMA) copolymer powder (15 w/v %) was redissolved in distilled water and then mixed with TDM with a mass ratio of 1:1. This composition was maximized with respect to TDM content to obtain the highest amount of dentinoconductive filler and correspond to the maximum amount of TDM powder that was allowed the formation of injectable formulations. Then riboflavin (12µM) as a photoinitiator was added to the mixture as recommended by Hyun H et al. [30] and Kim SH et al. [31]. As one distinguishing feature of riboflavin as a photoinitiator is that only minute amounts of riboflavin are required to activate the photocrosslinking process. The mixture was subsequently stirred for 5 minutes at room temperature in a dark glass bottle to avoid any premature polymerization due to the surrounding visible light irradiation [23, 32]. Glycine (0.5 mol%) as a coinitiator was added to the mixture and then stirred until a homogeneous solution was formed. To make the specimens, the mixture was injected into teflon molds (6 mm diameter, 4 mmm thickness) and photocrosslinked for 60 seconds using a light curing unit.

#### Grafting Optimization

The grafting efficiency (GE%), grafting percentage (GP%) and yield percentage of the obtained G-GMA/TDM grafted polymer were calculated depending on the following equations as follows [33]:

1 $$GP\;\left(\%\right) = \left(W1- W0\right)/W0 \times 100.$$

2 $$GE\;\left(\%\right) = \left(WG-GMA- WG\right)/GMA \times 100.$$

3 $$Yield\;\left(\%\right)=\left[W0/Wsolid\;content\right]\times100.$$

Where W1 is the weight of graft crosslinked hydrogels (G-MA) and W0 is the weight of native polymer (G) for GP%. WG-GMA is the weight of the vacuum-dried G-GMA macromonomer obtained; WG is the initial weight of neat G; and WGMA is the initial weight of GMA that has been introduced in the grafting reaction. While W0 is the dried gel weight and Wsolid content is the weight of the initial used polymer in the case of yield% [33].

#### Determination of swelling degree of crosslinked G-GMA/TDM hydrogel

The swelling or water uptake (%) of crosslinked G-GMA /TDM hydrogels (6 mm diameter, 4 mm thickness) was determined based on the equilibrium swelling theory state of hydrogels. The hydrogel was immersed in distilled water at pH 7.4 for 1–7 days and weighed at intervals (daily), with the hydrogel weight and volume increasing due to the swelling process. This step was repeated until no change in hydrogel weight was detected. This point is called the equilibrium swelling state of hydrogel, and then the equilibrium swollen hydrogel was freeze-dried. The water uptake (%) of hydrogels was given as follows [23]:

4 $$Water\;uptake\;\left(\%\right)=\left[\left(W_s-W_0\right)/W_0\right]\times100.$$

Where Ws is the weight of the swollen hydrogel and W0 is the weight of the dried hydrogel.

#### Hydrolytic degradation of crosslinked G-GMA/TDM hydrogel

The hydrolytic degradation of the disk shaped G-GMA /TDM hydrogels (6 mm diameter, 4 mm thickness) was determined by their weight loss (%) at 37 °C in a 50 mL glass beaker with constant shaking at 100 rpm. A 20 mL of phosphate buffer solution (PBS, pH 7.4 for 3 weeks) was added into glass beakers that contain G-GMA /TDM hydrogel disks. The hydrogel samples were incubated at 37 °C and the degradation media (PBS) was changed daily for three weeks. The change in sample weight due to hydrolytic degradation was weighed and measured at predetermined intervals.

The weight loss (%) was calculated by the given equation as follows [22]:

5 $$Weight\;loss\;\left(\%\right)=\left[\left(W_0-W_t\right)/W_0\right]\times100.$$

Where W0 is the original weight of hydrogel and Wt is the removed hydrogel from PBS and weighed at function of specific incubation intervals time.

#### Cytotoxicity test by MTT-assay

Cytotoxicity test was carried out by using Methylthiazolydiphenyl tetrazolium bromide (MTT) assay to quantify the exact nontoxic concentration of the prepared hydrogels which does not induce a toxic effect on the non-cancerous fibroblast cell line (WI-38b cells). An aliquot of 100.0 µl of 6 × 10^4^ cell/mL cells was seeded in each well of 96-well plates. The seeded plates were incubated for 24 h at 37 °C in humidified 5.0% CO_2_ or till semi-Confluency. After incubation, small cubic pieces of the hydrogels of different concentrations were added to the each well of the seeded 96 well plates. The prepared plates were incubated at the previous growth conditions for another 24 h. Formazan crystals were then dissolved in 200 mL/well of dimethyl sulfoxide, and the absorbance (A) was measured at 550 nm using a microplate reader (Urit 660; Guanxi, China). Cellular viability was measured using an MTT assay kit *(Promega*) according to the instructions of its manual, with wells containing cells in media as a control, without adding hydrogel extracts [34], as given in the equation:

6 $$Cell\;viability\;\left(\%\right)=\left({\mathrm A}_{\mathrm{test}}\right)/\left({\mathrm A}_{\mathrm{control}}\right)\times100$$

Where A _test_ is cells number after incubation and A _control_ is initial cells number before incubation.

#### Instrumental characterizations

*FTIR* type: (Shimadzu FTIR-8400S, Kyoto, Japan) was used. Transparent KBr-sample disk was prepared by crushing a polymer sample with infrared grade KBr and then pressing using force 105 N into the transparent disk. The FTIR spectrums were obtained by recording 64 scans between 4000–400 cm^−1^ with a resolution of 2 cm^−1^ [22].

^*1*^*H-NMR*, the proton nuclear magnetic resonance-spectrum was recorded by a NMR-DRX400 instrument with 300 MHz (BRUCKER, Karlsruhe, Germany). Typically, 25 mg of the polymeric sample was completely dissolved in 1.0 mL of deuterium oxide NMR-solvent (H_2_O_2_-d_6_), and then the sample was filleted using micro-filter before the measuring [22].

*SEM* type: (JEOL, JSM-6360LA, Tokyo, Japan) was used to investigate the surface morphology of crosslinked hydrogels. The hydrogel was first soaked in deionized water for 6 h to swell the interior channels and dispose of any impurities. The samples were dried by sudden freezing using liquid nitrogen and lyophilization at -90 °C under 0.5 mbar for 24 h. The dried hydrogel samples were then coated with gold using an ion sputter coater (model: 11430, USA, connected to a vacuum SPi module control model: 11425, USA) [22].

*TGA* instrument (Shimadzu TGA-50, Japan) with a heating rate of 40 °C/min under a flow of N2 up to 600 °C was used to test the thermal stability of gelatin and G-GMA/TDM hydrogels. Certain thermal kinetic parameters were determined from TGA results, such as T_onset,_ which defined as the temperature at the intersection of the baseline mass and the tangent drawn to the mass curve at the inflection point or point of the greatest rate of mass loss%. T_50_ is defined as the temperature at which the tested sample is thermally degraded and has reached 50% mass loss [33].

#### Statistical analysis

All experiments were performed with sample groups of at least three repeats (*n* = 3). All data are stated as mean ± standard deviation (SD). One-way analysis of variance (ANOVA) with Tukey’s Post Hoc Test was used for multiple comparisons. Statistical analysis was performed using IBM SPSS software package version 20.0. **(**Armonk, NY: IBM Corp**)**. Significance was accepted at *p* = 0.05.

## Results

### Synthesis of G-GMA/TDM hydrogels

**Fig. 1:**
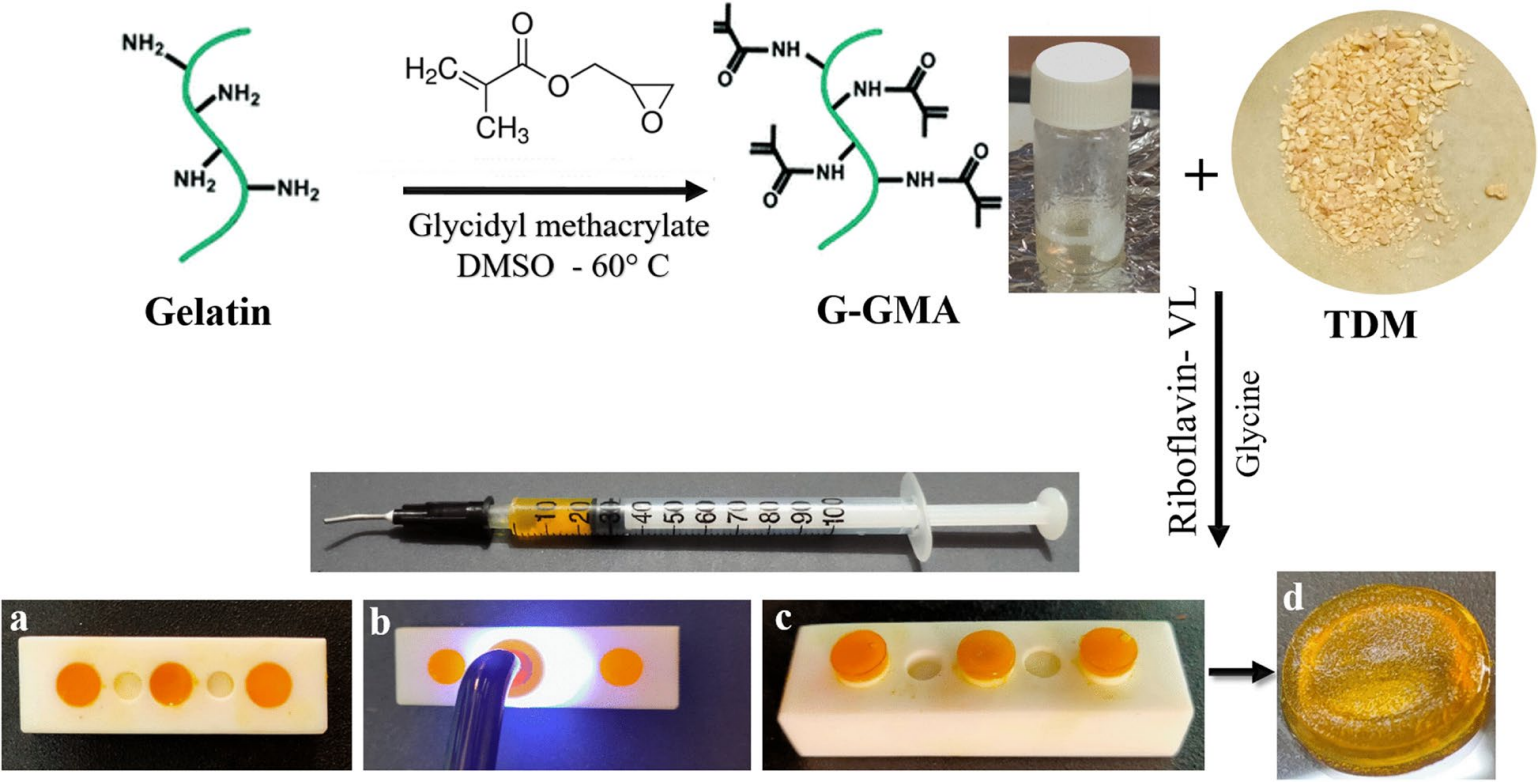
Shows an abbreviated schematic of the grafting reaction of gelatin with GMA and crosslinking of Gelatin-GMA /TDM hydrogel under visible light irradiation; (**d**) G-GMA/TDM hydrogel after light curing

Photopolymerized G-GMA/TDM macromonomers were synthesized by grafting gelatin with GMA via a trans-esterification reaction by introducing methacryloyl groups into gelatin chains. The Gelatin-GMA copolymer with TDM was crosslinked under visible-light irradiation at 330–470 nm for 60 s using a two-component photoinitiating system based on riboflavin as a photoinitiator and glycine as a coinitiator (Fig. 1).

### Grafting optimization of G-GMA/TDM hydrogels

#### Effect of GMA concentration and Effect of reaction time

**Fig. 2:**
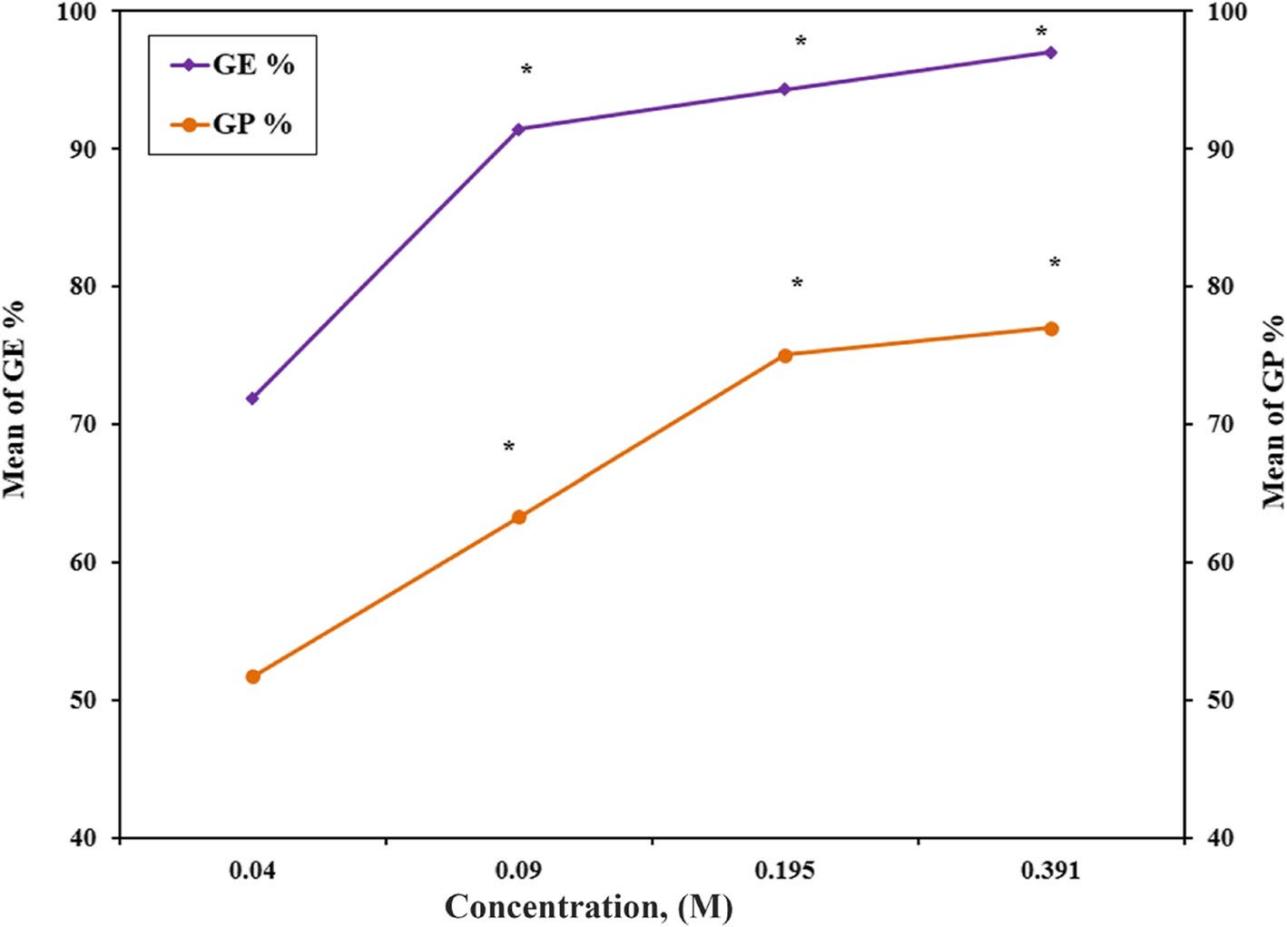
Effect of various GMA ratios (Gelatin/GMA): (1/0.048), (1/0.097), (1/0.195) and (1/0.391) M at 60 °C, on the formed G-GMA/TDM macro-monomer during the grafting reaction as a function of grafting efficiency and percentage

**Fig. 3:**
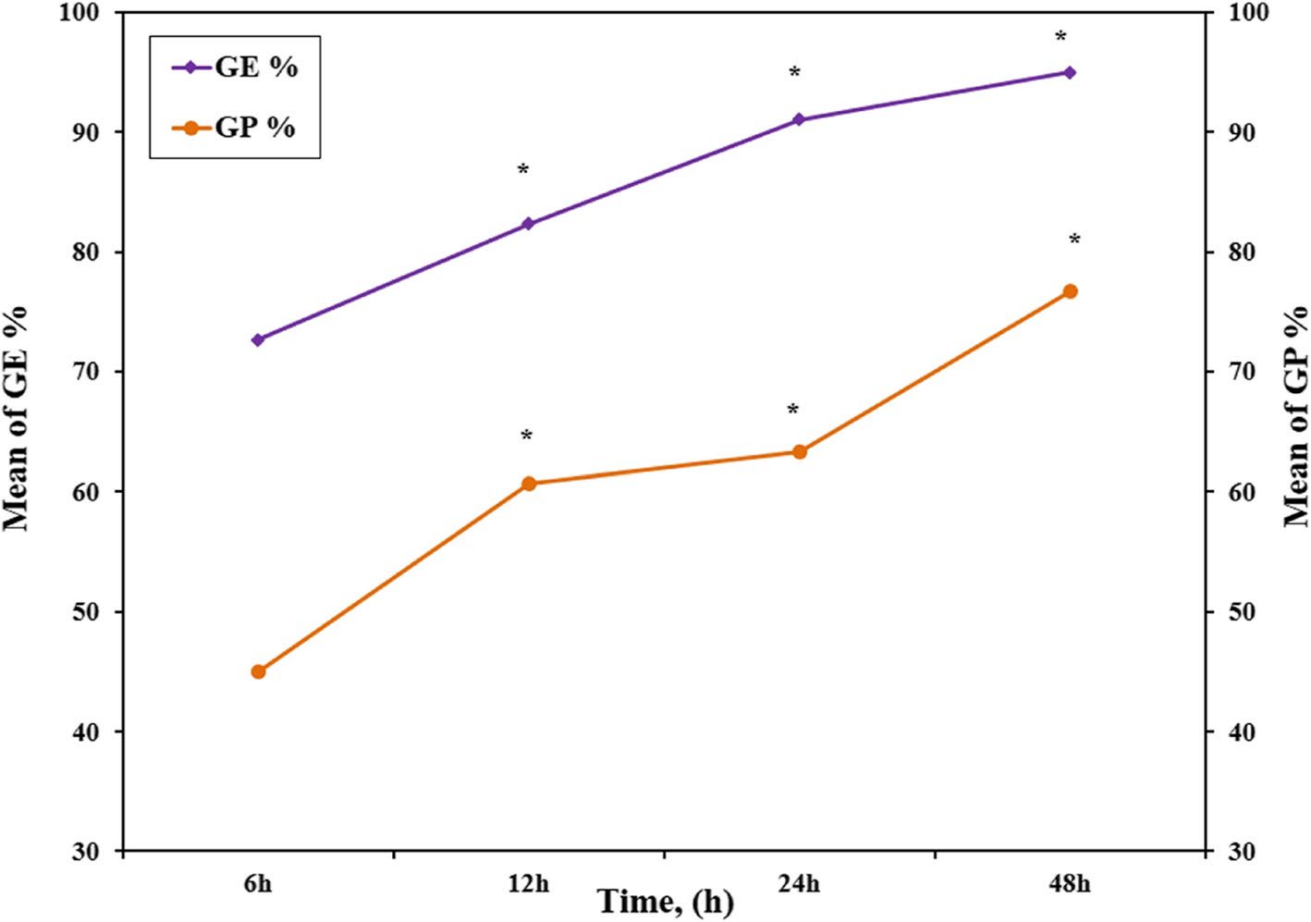
Effect of various reaction times (Gelatin/GMA): 6 h, 12 h, 24 h and 48 h at 60 °C, on the formed G-GMA/TDM macro-monomer during the grafting reaction as a function of grafting efficiency and percentage

The effect of different GMA concentrations and different reaction times on the grafting percent (GP%) and grafting efficiency (GE%) was exhibited in Figs. 2 and 3. It was observed that the grafting efficiency and grafting percentage showed the highest values of 97% and 77%, respectively, for Gelatin-GMA (1:0.391 M) and 95% and 76.7%, respectively, at a 48-h reaction time. Further, the yield percent of grafted methacrylate on gelatin was found to increase with increasing GMA concentration and reaction time. As Tables 1 and 2 demonstrate, the yield percent gradually increased from 80.7% to 96.3% for the GMA concentration and from 83.3% to 92% for the reaction time.

**Table 1:** Comparison between the G-GMA/TDM hydrogels with different GMA concentrations according to grafting percent (GP%), grafting efficiency (GE%), yield percent and cell viability

GMA Concentration	Yield percent (*n* = 3)	GE % (*n* = 3)	GP % (*n* = 3)	Cell viability (*n* = 3)
**0.04**	80.7^b^ ± 3.1	71.9^c^ ± 1.9	51.7^c^ ± 1.5	93.3^a^ ± 1.8
**0.09**	90^a^ ± 2	91.4^b^ ± 3.1	63.3^b^ ± 1.5	88.3^a^ ± 0.95
**0.195**	94^a^ ± 2	94.3^ab^ ± 1.5	75^a^ ± 1	69.5^b^ ± 0.38
**0.391**	96.3^a^ ± 2.5	97^a^ ± 1	77^a^ ± 1	51.1^b^ ± 3
**F**	24.164	93.312^*^	247.383^*^	34.720^*^
**P**	< 0.001^*^	< 0.001^*^	< 0.001^*^	0.003^*^

Data was expressed using Mean ± SD. *SD* Standard deviation

Mean with Common letters are not significant (i.e. Means with Different letters are significant)

*F* F for One way ANOVA test, Pairwise comparison bet. the Post Hoc Test (Tukey) was used in each of the two groups

*p p* value for comparing between the studied concentrations

^*^ Statistically significant at *p* ≤ 0.05

**Table 2:** Comparison between the G-GMA/TDM hydrogels with different reaction times according to grafting percent (GP%), grafting efficiency (GE%), yield percent and cell viability

Reaction time	Yield percent (*n* = 3)	GE % (*n* = 3)	GP % (*n* = 3)	Cell viability (*n* = 3)
**6h**	83.3^b^ ± 3.1	72.67^c^ ± 2.5	45^c^ ± 1	92.8^a^ ± 0.34
**12h**	85^ab^ ± 2	82.3^b^ ± 2.5	60.7^b^ ± 1.2	90.2^a^ ± 0.77
**24h**	90^ab^ ± 2	91^a^ ± 1	63.3^b^ ± 1.5	88.3^a^ ± 0.95
**48h**	92^a^ ± 4	95^a^ ± 0	76.7^a^ ± 1.5	67.95^b^ ± 2.6
**F**	6.010^*^	86.333^*^	289.317^*^	123.054^*^
**P**	0.019^*^	< 0.001^*^	< 0.001^*^	< 0.001^*^

Data was expressed using Mean ± SD. *SD* Standard deviation

Mean with Common letters are not significant (i.e. Means with Different letters are significant)

*F* F for One way ANOVA test, Pairwise comparison bet. the Post Hoc Test (Tukey) was used in each of the two groups

*p p* value for comparing between the studied Reaction time

^*^ Statistically significant at *p* ≤ 0.05

### Swelling degree of crosslinked G-GMA/TDM hydrogel

**Fig. 4:**
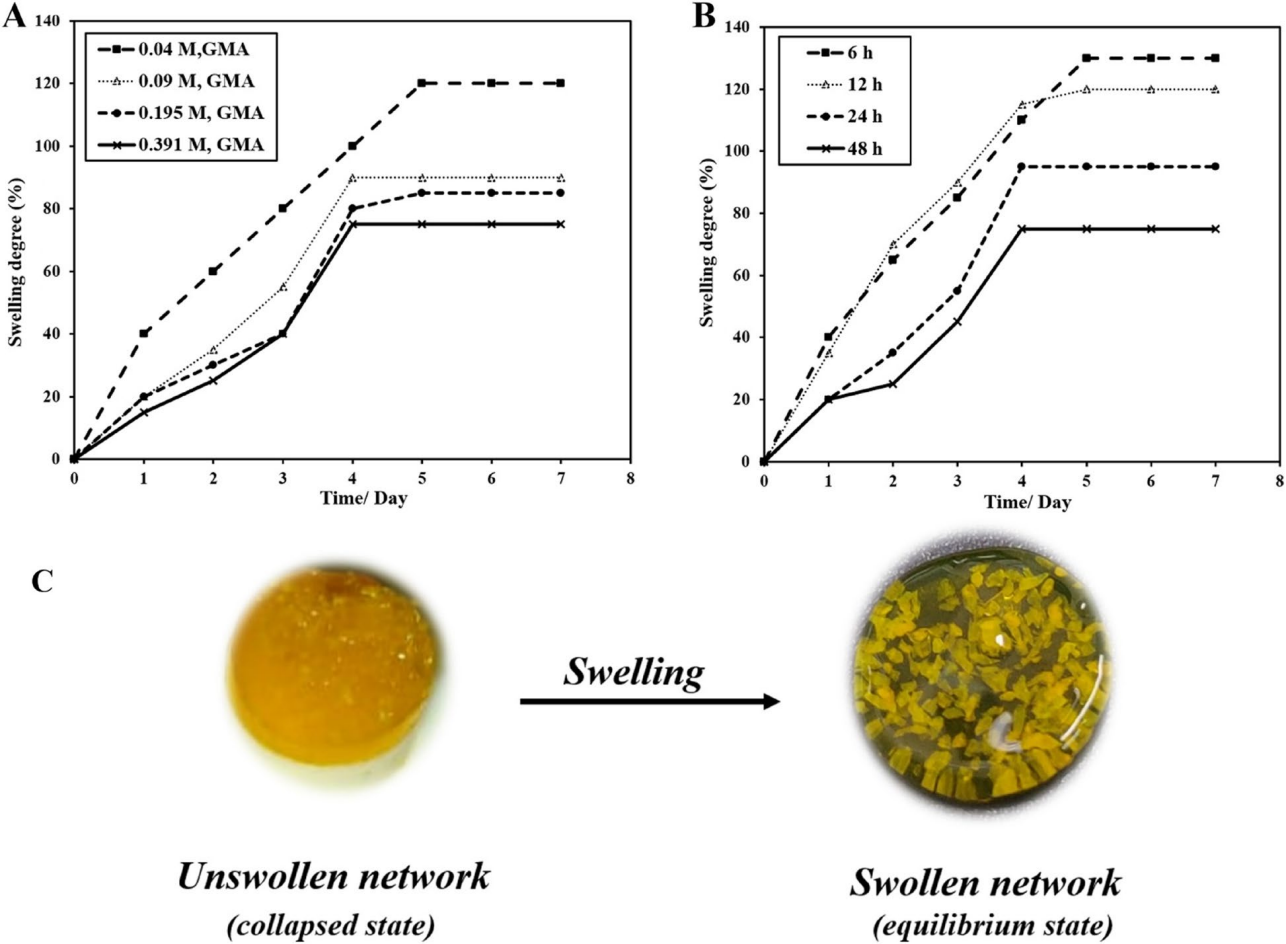
Swelling degree of G-GMA/TDM hydrogels with various GMA concentrations (**A**); various reaction times (**B**) after incubation in distilled water at 37 °C and (**C**) G-GMA/TDM hydrogel at equilibrium swelling state

Figure 4 shows the effects of the various GMA concentrations in macromonomers and the various reaction times of the Gelatin-GMA copolymer on the swelling ratio of crosslinked hydrogels. As shown, the swelling ratio of G-GMA/TDM hydrogels decreases gradually with the high GMA concentration of the copolymer, where hydrogels with GMA = 0.048 M swelled up to 120% and reached the equilibrium swelling state after 5 days. However, it decreases progressively with increasing the GMA contents, where hydrogels with GMA contents = (0.097, 0.195, 0.391 M) swelled up to 75–90% and reached equilibrium swelling after almost 4 days. Regardless of the reaction time, it was demonstrated that the swelling degree decreased gradually from 130 to 75% when the reaction time of prepared hydrogels increased from 6 to 48 h, respectively, as seen in Fig. 4B.

### Hydrolytic degradation of crosslinked G-GMA/TDM hydrogel

**Fig. 5:**
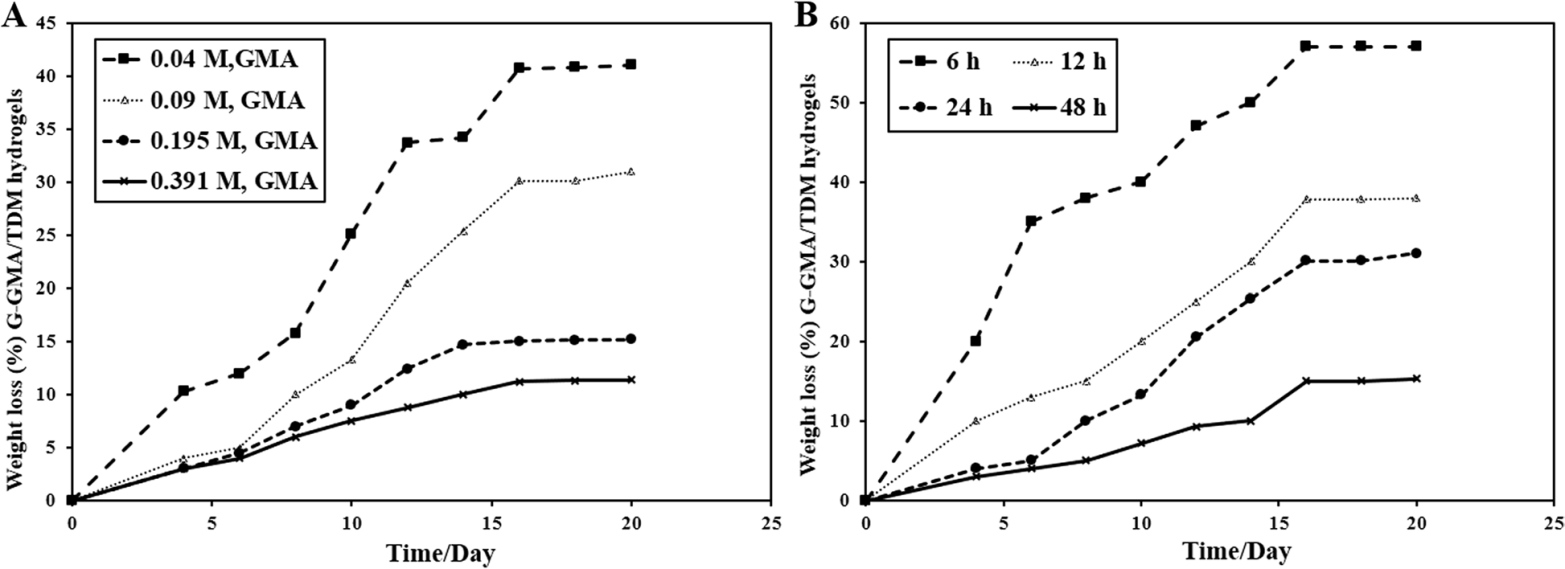
Representative of weight loss (%) of crosslinked G-GMA/TDM hydrogels as a function of different GMA concentrations in macromonomers (**A**) and different reaction times (**B**)

The hydrolytic degradation behavior of G-GMA/TDM hydrogels in PBS at pH 7.4 is presented in Fig. 5. The weight loss of prepared hydrogels with low GMA content (0.048 M) is about 41% after 16 days, whereas hydrogels with higher GMA contents of 0.097, 0.195 and 0.391 M have degraded slowly into 30%, 15% and 11%, respectively, after 16 days (Fig. 5A). Moreover, the weight loss of G-GMA/TDM hydrogels with a reaction time of 6 h is about 57% after 16 days, whereas hydrogels with prolonged reaction times of 12, 24 and 48 h have degraded slowly into 37.8%, 30.1% and 15%, respectively, after 16 days (Fig. 5B).

### Cytotoxicity test by MTT-assay

**Fig. 6:**
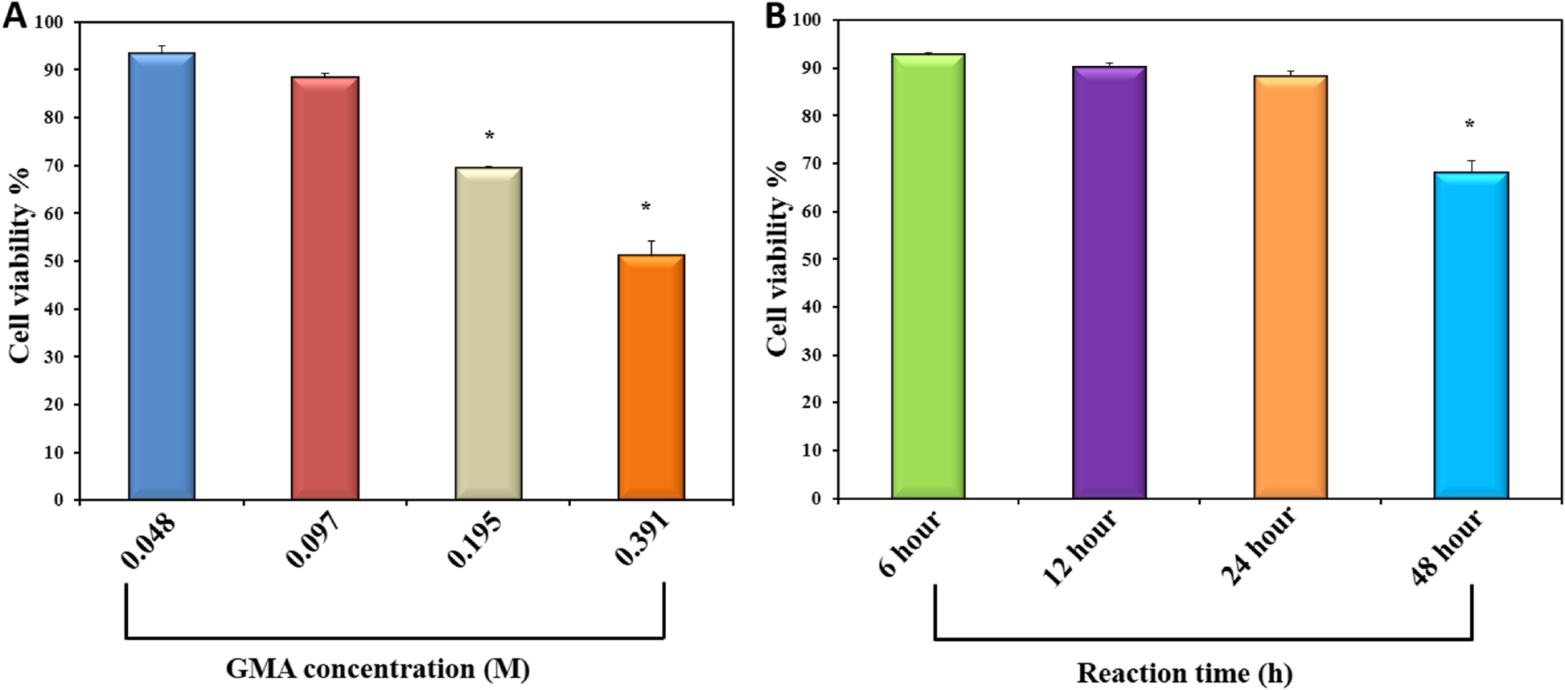
Cytotoxicity test of photopolymerized G-GMA/TDM with various GMA concentrations (**A**); various reaction times (**B**). The data represented the mean and the average of triplicate was taken

The cell viability of crosslinked G-GMA/TDM hydrogels with two different parameters (GMA concentration and reaction time) using the MTT assay on the fibroblast cell line (WI-38b cells) is presented in Tables 1 and 2 (Fig. 6A, B). As seen, Cell viability (%) was found to be highest in 0.04 and 0.09 M GMA (93.3% 1.8 and 88.3% 0.95, respectively), with a statistically significant difference compared to the other GMA concentrations (0.195 and 0.391 M) (69.5% 0.38 and 51.1% 3).In terms of reaction time, results confirm biocompatibility for all hydrogels with different reaction times (6 h, 12 h, and 24 h), with the exception of the hydrogel with a 48 h reaction time, where cell viability is 92.8% 0.34, 90.2% 0.77, 88.3% 0.95, and 67.95% 2.6, respectively.

### Characterization of G-GMA/TDM macromonomers and hydrogels

#### Fourier transform infra-red spectroscopy and ^1^H-NMR spectra

**Fig. 7:**
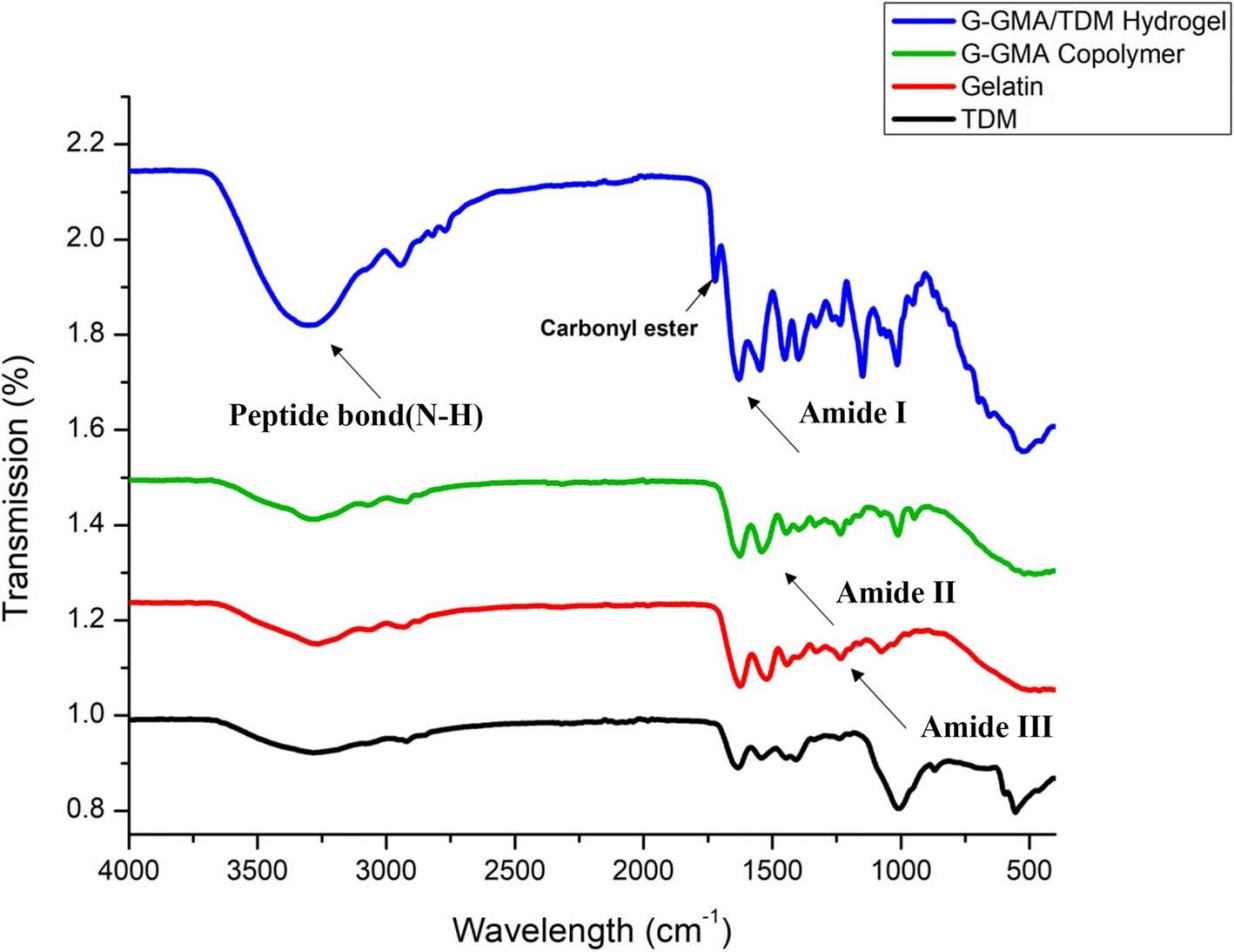
FTIR spectrum of TDM, Gelatin, G-GMA copolymer, G-GMA/TDM crosslinked hydrogel with a molar ratio (1/0.097). IR (KBr): ѵ = 1722.5 cm.^−1^ (O–CO–O, carbonyl ester group)

FTIR spectra showed the grafting reaction between gelatin and GMA forming G-GMA/TDM crosslinked hydrogel, as shown in (Fig. 7**).** It was noticed that a band of a carbonyl ester group at ν 1722.5 cm − ^1^ exists clearly only with crosslinked G-GMA/TDM hydrogel, where its absorbance intensity increases strongly and gradually with the crosslinking degree. However, this band neither appears in pure Gelatin and TDM nor in G-GMA copolymer (i.e., uncrosslinked polymer). Moreover, the spectra of Gelatin, G-GMA copolymer and G-GMA/TDM crosslinked hydrogel showed a broad peak at ν 3300 cm^− 1^ associated with the stretching of the -OH and peptide bonds (mainly N–H stretching). The peaks at ν 1624, 1521 and 1235 cm^− 1^ observed in gelatin were due to the presence of amide I amide II and amide III, respectively; and ν 3266 cm^− 1^ related to N–H stretching vibration [25, 35]. Similar peaks were found in the Gelatin, G-GMA copolymer and G-GMA/TDM crosslinked hydrogel, which inferred that after the reactions no such chemical changes occurred in the amide bonds.

**Fig. 8:**
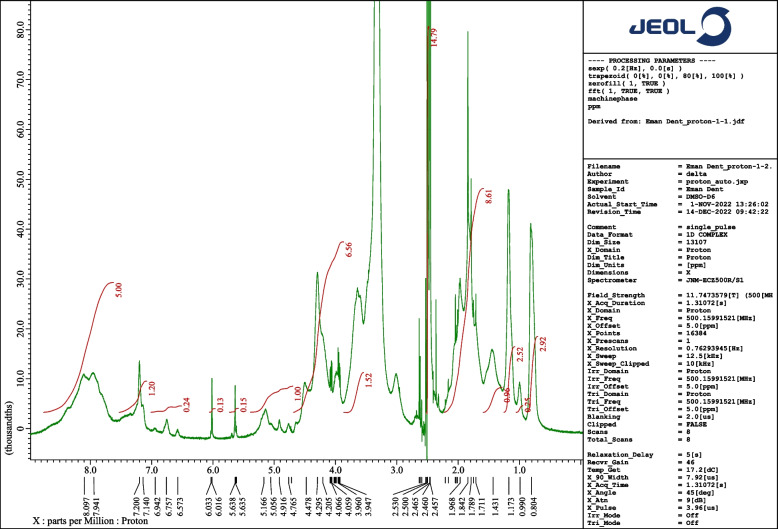
^1^H-NMR spectrum of G-GMA macro-monomer with a molar ratio (1/0.097)

The successful coupling of GMA as a side chain in the Gelatin structure was evidenced by the ^1^H-NMR spectra of the resultant copolymer. The spite carbonyl ester group, which is responsible for the crosslinking performance of G-GMA/TDM hydrogel could not be detected by ^1^H NMR spectra; however, this spectrum was utilized to determine the coupling reaction resulting in the Gelatin-GMA copolymer (Fig. 8). Where, the signals of methyl protons of methacrylate groups are observed at δ 1.84 ppm which indicates the successful functionalization of gelatin. Furthermore, the two signals that were observed at δ 6.01 and 5.6 ppm for the sample are due to the vinyl protons in methacrylate groups. Also at the range δ 3.9- 4.06 ppm, two multiple signals are observed, each equivalent to one proton corresponding to the O-CH2 group. Taken together, the ^1^H-NMR spectrum of the modified G-GMA grafted macro-monomer shows the appearance of -CH3 and vinyl protons signals, which confirm the successful reaction of GMA to the gelatin biopolymers.

### Morphological investigation by SEM

#### Effect of GMA concentration and their reaction time on the surface morphology of crosslinked G-GMA/TDM hydrogels

**Fig. 9:**
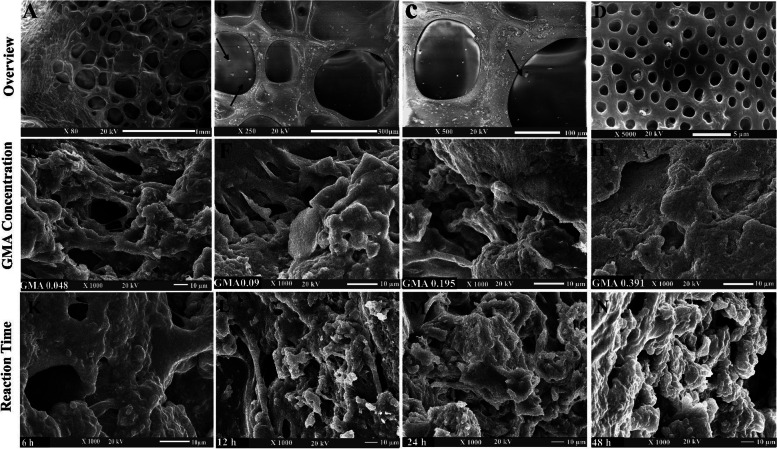
SEM images of the freeze-dried crosslinked G-GMA/TDM hydrogels were shown as an overview image of the hydrogel (**A**); (**B** and **C**) arrows showed TDM impregnated within G-GMA pores and showed dentinal tubules of TDM sufficiently exposed (**D**) (original magnification × 80, × 5000); with various GMA concentrations as (**E**,** F**,** G**,** H**) (0.048, 0.097, 0.197, 0.391 M, respectively) (original magnification × 1000); and various reaction times as (**K**,** L**,** M**,** N**) (6 h, 12 h, 24 h, 48 h, respectively) (original magnification × 1000)

The microstructure of lyophilized crosslinked G-GMA/TDM hydrogels with various GMA concentrations and various reaction times was characterized by SEM and shown in Fig. 9. An overview photograph of the hydrogel at low magnification (× 80) reveals a well-defined 3-D interconnected porous microstructure with TDM impregnated within its pores (Fig. 9A). Furthermore, scanning electron microscopic characterization of TDM showed dentinal tubules sufficiently exposed and loosened fiber bundles of intertubular and peritubular dentin after specific EDTA treatment at × 5.000 (Fig. 9D). As seen, a heterogeneous morphological structure showing randomly, evenly distributed polyhedral pores were observed with both cryogels with low GMA concentrations and short reaction time. However, the pore walls of G-GMA/TDM cryogel surfaces were more compact, smooth, uniform, and dense compared to high GMA concentrations and prolonged reaction time. Additionally, it can be observed that the porosity increased with decreasing GMA hydrogel content and reaction time. As, G-GMA/TDM hydrogels with a higher GMA content and prolonged reaction time exhibited a smaller pore size and a denser and compacted surface hydrogel morphology.

### Thermal analysis by TGA

**Fig. 10:**
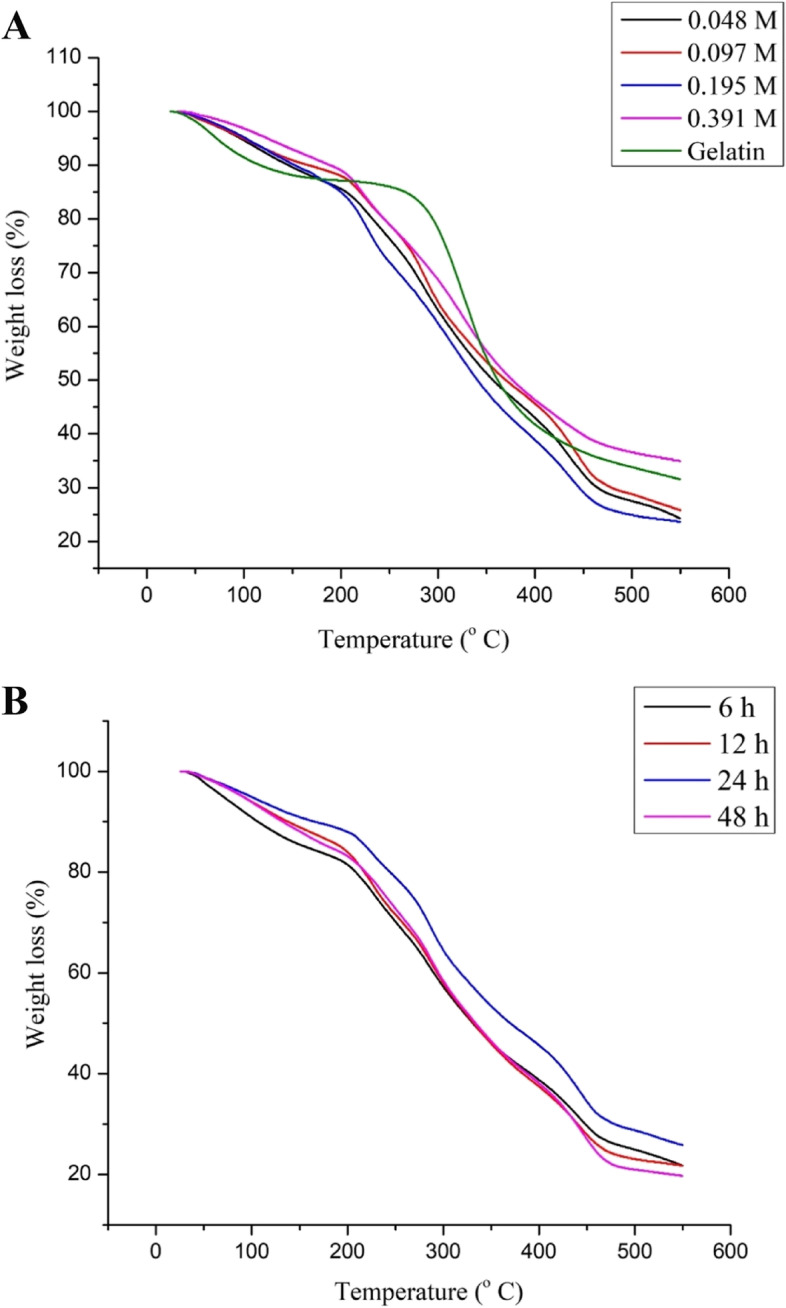
TGA thermograms curves of G-GMA/TDM crosslinked hydrogels with different GMA ratios (**A**); and different reaction times (**B**)

The thermal properties of pure gelatin and crosslinked G-GMA/TDM dried hydrogels have been studied by TGA. The thermogram results of the formed composite hydrogels revealed that the T_onset_ of the pure gelatin is 71.29 °C and that it lost 50% of its original weight T_50_ at 361.5 °C (Fig. 10). However, G-GMA/TDM hydrogels exhibit different weight loss behavior, due to the enhanced thermal stability of hydrogels as compared to pure gelatin, whereas the T_onset_, T_50_ values, and weight loss of G-GMA/TDM hydrogels were obviously enhanced by increasing the GMA contents in hydrogels. As T_onset_ and T_50_ values increased gradually from 95.77 to 122.73 and 356 to 375.8 °C, respectively, with increasing GMA concentrations in hydrogels. Regardless of the reaction time, it was observed that as the reaction time increased, the thermal stability of the resultant increased until 24 h later; they returned to reduce with prolonging the reaction time until 48 h. As T_50_ for the prepared scaffold with different reaction times (6, 12, 24, 48 h) is 330.56, 331.56, 369.5, and 333.56 °C, respectively.

## Discussion

Photopolymerizable materials are getting popular with extended use and applications with regard to the field of tissue engineering. The visible-light crosslinking method has been intensively utilized in dental clinics for in situ hydrogel material preparation. In the current study, a novel scaffold made from gelatin-treated dentin matrix hydrogel with the capability of being light-cured using a natural photoinitiating system composed of riboflavin and glycine was experimentally prepared and characterized for potential use in the regeneration of the dentin-pulp complex to maintain pulp vitality. Glycidyl methacrylate is a key ingredient in the production of light-cured hydrogels. Both its concentration and reaction time have a significant influence on the properties of prepared hydrogels, such as grafting optimization, swelling degree, degradation rate, biocompatibility, surface morphology, and thermal stability. As a result, the current study specifically investigated the effect of GMA concentration and reaction time on the various properties of gelatin-treated dentin matrix hydrogel in order to select the optimal ratio and reaction time for initiating the in vivo attempts of gelatin-treated dentin matrix hydrogel as a novel photocrosslinkable pulp capping agent for dentin regeneration in dog's teeth.

It was anticipated that increasing the final weight of the grafted hydrogel over the initial weight of the polymer component would demonstrate the grafting process. Evidently, the grafting percent and efficiency rise as the degree of substitution (DS) or grafting degree rises. As reported by Kim et al. [36], the DS of the prepared hydrogel by methacrylate increased with an increase in both GMA concentration and reaction time. This fact was linked to the rise in DS, which was also responsible for a greater degree of crosslinking in the hydrogel matrix. According to Crispim et al. [37] and Kamoun et al. [38], a higher DS denotes a significant amount of methacrylate groups connected to the polymer chains, which results in an increase in initiated sites on gelatin and a large number of monomer moieties attached. Furthermore, as the concentration of GMA and reaction time increased, the yield percent of grafted methacrylate on gelatin gradually increased. This effect might be caused by the gel phase's compactness, which might limit the mobility of polymeric chains and prevent further crosslinking reactions. This is comes in accordance with those of Pitarresi et al. [39] and Kim et al. [36].

One of the most crucial hydrogel features that have to be addressed is swelling, since it affects the surface morphology of hydrogels, the rate of releasing bioactive molecules and the hydrolytic degradation rate through the scaffold. It's interesting to note that the swelling degree (SD) values have drastically increased as a result of lower GMA ratios and they gradually decrease as the GMA content increases. This behaviour was attributed to hydrogels with low macromonomer contents (i.e., 0.048 M of GMA) possessing larger pore sizes than those with high GMA contents, as shown in Fig. 9. The hydrogel's 3D network with higher GMA contents became more compact and tight, which would limit the number of free hydroxyl groups available. As a result, it is much more difficult for water molecules to penetrate the hydrogel network, which results in a lower swelling degree. The increase in crosslinking degree restricts the polymeric matrix expansion, and subsequently less water is absorbed [22]. This finding is entirely consistent with the grafting and dextran-MA hydrogel formation results [36], showing dextran-methacrylate hydrogels displayed a wide range of swelling, ranging from 67 to 227%. As methacrylate substitutions in hydrogels decline, their swelling rises and the hydrogel matrices expand. Tiwari A et al. [40] studied the swelling properties of the photopolymerizable guar gum–methacrylate hydrogels (GG–MA), showed that the swelling ratio initially increased with decreasing methacrylate gel content, and that swelling was inhibited by the more rigid network at higher cross-linking density. This could be attributed to the increasing hydrophilicity of the GG–MA hydrogels resulting from a relatively lower number of hydrophobic poly(methacrylate) kinetic chains formed as a result of the lower degree of cross-linking. Furthermore, poly (γ-glutamic acid) hydrogel exhibited a lower swelling degree from 0.032 to 0.016 as the concentration of crosslinkers increased from 2 to 10% [41]. As the reaction time of prepared hydrogels increased, the degree of swelling significantly diminished. This implies that prolonging the reaction time increases the molecular weight [42], which improved the grafting optimization of G-GMA/TDM hydrogels as there is a linear relation between molecular weight and grafting optimization as mentioned by Yasuhiko Onishi et al. [43]. As grafting optimization increased, the substitution degree of G-GMA/TDM hydrogels and the crosslinking degree increased as well, and the swelling degree decreased [36, 44]. Additionally, this inverse relationship between the swelling degree, prolonging the reaction time and the substitution degree is consistent with the previous swelling results that were reported by Kamoun et al. [22].

The in vitro hydrolytic degradation or weight loss (%) of G-GMA/TDM hydrogels with different concentrations of macromonomers (GMA contents) and different reaction times was investigated in terms of weight loss for 3 weeks in PBS solution (pH 7.4 at 37 °C). Because gelatin is an excellent hydrophilic polymer and because of its porous structure after swelling Fig. 4C, it absorbs PBS buffer. Subsequently, the degradation of G-GMA/TDM hydrogels takes place through bulk erosion at the surface and interior, simultaneously. The weight loss rate of G-GMA/TDM hydrogels with low GMA contents was faster than that with high GMA contents. These results revealed that GMA contents have slowed down the hydrolytic degradation rate and weight loss (%) of hydrogels, which ranged from 41—11%, respectively, with increasing GMA contents. This is owing to high crosslinking density hydrogels related to high GMA contents, which increase the stability of the hydrogels and hinder the degradation rate. These outcomes are entirely consistent with the results of Kamoun et al. [26], who proved that the rate of degradation of PVA-g-GMA hydrogel became faster with decreasing GMA contents compared to the degraded hydrogels with high GMA contents. Moreover, Baier Leach et al. [21], who fabricated photocrosslinked GMA hyaluronic acid hydrogels (GMHA) found the degradation of 5% methacrylated GMHA gels (10.6 ± 2.0%) was significantly faster than that of 7% methacrylated GMHA gels (4.6 ± 0.8% *p* < 0.001), and the degradation of 7% was faster than that of 11% methacrylated GMHA gels (0.7 ± 0.3%; *p* < 0.0001). In terms of reaction time, the weight loss of G-GMA/TDM hydrogels with a short reaction time (6 h) is greater. The degree of crossing linking and the substitution degree of G-GMA/TDM hydrogels increase as reaction time increases, as was previously mentioned [36], and the stability of the hydrogels increases; therefore, the degradation rate will be low. It was concluded by Baghban et al. [7] that increasing the GMA concentrations and reaction time to the optimum values enhanced the physicochemical properties of the LED-curable methacrylated gelatin hydrogel.

Toxicology testing is thought to be an effective aspect of dental biomaterials. Ideal biomaterials shouldn't induce adverse reactions or release any hazardous compounds. According to ISO 10993–5 (E) [45], results revealed that the prepared scaffolds with 0.04 and 0.09 M GMA concentrations were biocompatible, as the cell viability (%) remained higher than 70%. The significant decrease in metabolic activity of cells treated with 0.195 and 0.391 M GMA could be attributed to high GMA content hydrogels exhibiting low protein adsorption and some toxic effects, whereas low GMA content hydrogels exhibited the highest safety on human fibroblast cells. This finding is consistent with the findings of Derya Sürmeliolu et al. and Beltrami R et al. [46, 47], who found that GMA has a concentration-dependent cytotoxic effect on fibroblast cells. Hong H et al. [48] showed that the fibroblast cell line NIH3T3 proliferation rate increased due to the elimination of residual unreacted GMA from modified silk fibroin with glycidyl methacrylate (Silk-GMA) by dialysis period. An optimal crosslinking reaction time produces a hydrogel with good physical properties via adequate crosslinking while ensuring optimum cell survival by lowering exposure to toxic reaction products [49]. All hydrogels with different reaction times (6 h, 12 h, and 24 h) were biocompatible except for the hydrogel with a 48 h reaction time, as gelatin is a cationic polymer that could have influenced the findings. According to Shuangcheng Tang et al. [42] and Bryn D. Monnery et al. [50], the molecular weight of cationic polymers will increase as a function of time, and materials with higher molecular weights are more toxic.

Additionally, the surface morphology of the G-GMA/TDM cryogels with different GMA concentrations and different reaction times was prepared by cryo-fixation and cryo-fracturing techniques [51]. The improvement of the morphological surface of hydrogels with high GMA concentration and prolonged reaction time was attributed to the fact that the higher the crosslinking degree, the higher the substitution degree copolymer contains high levels of photocurable methacrylate groups, allowing the formation of hydrogels with high crosslinking density and uniform surface structure. These results are fully consistent with those obtained by Kamoun and Menzel et al. [22], who revealed that the high substitution degree of pullulan-HEMA hydrogels crosslinked by carboxylated camphorquinone folic acid coinitiator under visible light exhibited dense and compacted surface hydrogel morphology compared to the low DS copolymer. Further, Tiwari A et al. [40] examined the morphology of the photopolymerizable guar gum–methacrylate hydrogels (GG–MA), which exhibited a 3-D interconnected open pore microstructure. As the porosity increased with decreasing methacrylate hydrogel content. Thus, guar gum–methacrylate.hydrogels with a higher methacrylate gel content resulting from the higher methacrylation degree percent of the corresponding GG–MA macromonomers had a smaller pore size and a higher pore density.

The thermal gravimetric analyses of pure gelatin and G-GMA/TDM dried hydrogels have been described according to the thermal weight loss (%) and T_onset_ values. The overall TGA parameters have improved with increasing GMA content and reaction time until 24 h, and afterwards they returned to a lower level with prolonging the reaction time until 48 h. This behavior could be attributed to the grafting of GMA onto gelatin and the prolonged reaction time, which enhanced the thermal stability of the formed hydrogel due to the high crosslinking density and compactness of hydrogels, as the enhanced thermal properties of the G-GMA/TDM with high GMA concentration and 24 h reaction time synthesized hydrogels suggested an effective methacryloyl conjugation leading to a greater amount of covalent crosslinking density [7, 33, 35]. These result are consistent with Kamoun et al. [38] who studied PVA-g-GMA hydrogels with different GMA concentrations (0, 0.025, 0.05, 0.07, 0.09, 0.15 and 0.25 M), all TGA determined parameters of PVA-g-GMA hydrogels have thermally improved due to the increase of the ratio of GMA until 0.09 M later, they return to reduce again with high ratios of GMA at 0.125 and 0.25 M.

According to our research results, the ratio and reaction time of GMA (0.097 M ~ 2 mL for 24 h) have been selected as the optimal ratio and optimal reaction time for initiating the in vivo attempts of G-GMA/TDM as a novel photocrosslinkable pulp capping agent for dentin regeneration in dog's teeth, which will be the second part of this research. As G-GMA/TDM (0.097 M ~ 2 mL for 24 h) synthesized hydrogel, a biocompatible scaffold, this is a key consideration for selecting pulp capping material that is not cytotoxic on dental pulp cells, and another significant concern is an improved swelling degree, as it is a crucial point in preparing hydrogel for dentin regeneration that allows the feasible release of signaling molecules along with calcium and phosphate from the scaffold for tissue mineralization,, as well as offer required features for improving surface morphology, ideal grafting reaction conditions with a convenient degradation rate with better thermal stability.

## Conclusions

The novel gelatin-GMA/treated dentin matrix hydrogels are based on a riboflavin visible-light induced system and glycine as a coinitiator for the first time with riboflavin. The synthesis procedure of an uncrosslinked Gelatin- GMA copolymer was described in detail and confirmed via NMR spectra, while the crosslinking of G-GMA/TDM hydrogel was determined by IR spectroscopy. The concentration of GMA and its reaction time were utilized to adjust the properties of the resultant crosslinked hydrogels. As a result, using 0.097 M of GMA content and prolonging its reaction time for 24 h resulted in high crosslinked density hydrogels, high thermal stability, compacted surface morphology hydrogels, and biocompatible hydrogels. Thus, visible-light photocrosslinked G-GMA/TDM hydrogels could be employed as novel biomaterial and scaffold in dentin engineering.

### Supplementary Information

**Additional file 1.** Characterization Data 1.

**Additional file 2.** Characterization Data 2.

**Additional file 3.**

## Data Availability

All data generated or analyzed during this study are included in this published article and its Additional files 1 and 2.

## Abbreviations

TDM: Treated dentin matrix
DPC: Direct pulp capping
G: Gelatin
GMA: Glycidyl methacrylate
VL: Visible light
ECM: Extra cellular matrix
EDTA: Ethylenediaminetetraacetic acid
PBS: Phosphate buffer saline
GE: Grafting efficiency
GP: Grafting percentage
MTT: Methylthiazolydiphenyl tetrazolium
SD: Standard deviation
